# Geographic and Temporal Patterns of Screening for Breast, Cervical, and Colorectal Cancer in the US, 1997-2019

**DOI:** 10.1001/jamanetworkopen.2025.37905

**Published:** 2025-10-17

**Authors:** Pranoti Pradhan, Hari S. Iyer, Timothy R. Rebbeck

**Affiliations:** 1 Department of Epidemiology, Harvard T.H. Chan School of Public Health, Boston, Massachusetts; 2 Division of Population Sciences, Dana-Farber Cancer Institute, Boston, Massachusetts; 3 Section of Cancer Epidemiology and Health Outcomes, Rutgers Cancer Institute, New Brunswick, New Jersey

## Abstract

**Importance:**

Despite guidelines and recommendations, cancer screening remains low and variable across geographic and sociodemographic groups, contributing to persisting disparities.

**Objective:**

To analyze geographic and temporal patterns of county-level cancer screening prevalence for breast, cervical, and colorectal cancer over a 22-year period in the US and examine sociodemographic factors associated with screening clusters.

**Design, Setting, and Participants:**

This cross-sectional study used an ecological panel design using county-level screening prevalence as the unit of analysis. Data were collected from US mainland counties (ie, excluding Alaska, Hawaii, and Puerto Rico) from 1997 to 2019, with prevalence estimated over 3- to 5-year periods. Data were analyzed from 2024 to 2025.

**Exposures:**

County-level screening prevalence was estimated from the Behavioral Risk Factor Surveillance System and the National Health Interview Survey from 1997 to 2019. Socioeconomic and demographic characteristics were estimated from the 2000 US Census and linked with county and geographic clusters of screening.

**Main Outcomes and Measures:**

Spatial autocorrelation analyses, including Global Moran* I* and bivariate local indicator of spatial autocorrelation, were conducted to assess geographic clustering of county-level cancer screening prevalence over time. A queen contiguity matrix defined neighboring counties, and permutation tests evaluated statistical significance.

**Results:**

Data from 3142 counties were assessed. Spatial analyses of mammography, Papanicolaou test, and colorectal cancer screening in the contiguous US from 1997 to 2019 revealed consistent geographic clustering, with high screening prevalence in the Northeast and lower prevalence in the Southwest. Spatial autocorrelation, measured by Global Moran I, declined over time. For example, the distribution of mammography screening became 83% more uniform in more recent years (eg, Moran *I* = 0.57 in 1997-1999 vs Moran *I* = 0.10 in 2017-2019). Similarly, Papanicolaou test screening became more uniform in more recent years (eg, Moran *I* = 0.44 in 1997-1999 vs Moran *I* = 0.07 in 2017-2019). Bivariate local indicator of spatial autocorrelation analyses identified clusters of persistently high and low screening, with sociodemographic characteristics associated with clusters that changed from low to high. For example, for both Papanicolaou test and mammography, areas that changed from low to high were more likely to be low socioeconomic status, suggesting improvements in disadvantaged areas in 2017 to 2019 vs 1997 to 1999. Disadvantaged areas were most likely to experienced persistently low screening, particularly for breast or colorectal cancer.

**Conclusion and Relevance:**

These findings suggest that despite increasing screening overall, which led to reduced geographic variation in screening, local clusters of high and low screening persisted in the Northeast and Southwest US, respectively. Future studies could incorporate county-level health care access characteristics to explain why areas of low screening did not catch up to optimize cancer screening practices.

## Key Points

**Question:**

How did geographic patterns of county-level cancer screening prevalence for breast, cervical, and colorectal cancer change from 1997 to 2019 in the US, and what sociodemographic factors were associated with screening clusters and trends?

**Findings:**

This cross-sectional study using data from 3142 counties from 1997 to 2019 found persistent geographic clusters, such as high screening prevalence in Northeast and lower prevalence in Southwest. Socioeconomic disadvantage was correlated with clusters of persistently low screening prevalence but also with some areas that increased screening prevalence over time.

**Meaning:**

This cross-sectional study found that despite overall screening increases, some clusters of low screening persisted and were associated with socioeconomic disadvantage.

## Teaser

This cross-sectional study examines geographic and temporal patterns of county-level screening prevalence for breast, cervical, and colorectal cancer from 1997 to 2019 in the US and describes sociodemographic factors associated with screening clusters.

## Introduction

Cancer is the second leading cause of death in the US.^1^ In 2025, breast cancer accounted for 42 170 deaths, colorectal cancer for 52 900 deaths, and cervical cancer for 4320 deaths.^1^ To reduce cancer-related mortality, the US Preventive Services Task Force (USPSTF) recommends biannual mammography screening for women aged 40 to 74 years; colorectal cancer screening for adults aged 45 to 75 years with varying frequencies depending on the testing method; and cervical cancer screening every 3 years with cervical cytology (Papanicolaou test) for women aged 21 to 29 years, followed by human papillomavirus primary screening every 5 years for women aged 30 to 65 years.^2,3,4^ These recommendations are based on results from randomized clinical trials and/or cohort studies that have demonstrated a significant reduction in cancer mortality or a decrease in the stage at which cancer is diagnosed when appropriately followed and implemented.^2,3,5^ Despite these recommendations, screening for these cancers remains below national targets. As of 2021, among the population eligible for screening, 75.7% were screened for breast cancer, 72.2% for colorectal cancer, and 75.2% for cervical cancer—falling short of the Healthy People 2030 goals of 77.1%, 74.4%, and 84.3%, respectively.^6^ Access barriers, including lack of insurance, regular health care practitioner visits, and knowledge about screening, likely contribute to these gaps in screening.^7,8,9^

A crucial but understudied aspect of health care access is how the uptake of these breast, colorectal, and cervical cancer screening tests varies across time and geography. Availability, affordability, and accessibility likely contribute to disparities in screening, leading to differences in health outcomes across populations.^10,11,12,13^ Sociodemographic and sociopolitical factors may impose barriers on individuals from racialized minority groups, including those who self-identify as Black or Hispanic, thereby widening gaps in screening rates.^12,14^ In some settings, such as the Veterans Health Administration, racial and socioeconomic disparities in cancer mortality are attenuated, highlighting the importance of optimal health care access to narrow cancer disparities.^15,16^

Few studies have investigated geographic and temporal patterns of screening. Most research to date has been limited to single states or regions, a single intervention (eg, mammography), or generally examined time trends and static maps, rather than considering spatial statistical structure.^17,18,19,20,21^ In addition, most studies have evaluated availability of screening services (eg, whether testing is available at facilities close to individuals at risk) rather than the actual uptake of screening tests.^17,18,19,20,21^

To address this gap in the literature, we performed one of the first nationwide geographic and temporal examinations of county-level prevalence of screening for breast, cervical, and colorectal cancer over a 22-year period in the US. These cancer screening strategies were chosen based on the consensus from major public health bodies that screening benefits outweigh harms.^22^ Our goal is to describe changes in geographic clusters of screening prevalence and examine sociodemographic factors associated with clusters and time.

## Methods

This cross-sectional study was conducted as a secondary analysis of deidentified public databases. Studies involving secondary analysis of deidentified public use datasets do not require prior institutional review board approval or informed consent, per the National Institute of Health Office of Human Subjects Research and 45 CFR 46.104.^23^ This study is reported following the Strengthening the Reporting of Observational Studies in Epidemiology (STROBE) reporting guideline.

### Study Setting and Data

This ecological panel study of screening prevalence considered each US mainland county as the unit of analysis. County was chosen as the unit of analysis to allow stable geographic comparisons over time, and because many public health administrative policies occur at county level. We excluded Alaska, Hawaii, and Puerto Rico because counties share few neighbors, a condition for use in spatial statistical analysis. We identified geographic clusters of screening use in 3- to 5-year increments from 1997 to 2019.

County-level screening prevalence was obtained from the publicly available Small Area Estimates for Cancer-Relates Measures (SAE) from the National Cancer Institute (NCI).^24,25^ We obtained county-level estimates of prevalence of mammography (from 1997-1999, 2000-2003, 2004-2007, 2008-2010, 2011-2016, and 2017-2019), Papanicolaou test (from 1997-1999, 2000-2003, 2004-2007, 2008-2010, 2011-2016, and 2017-2019), colonoscopy (from 2011-2016 and 2017-2019), colorectal cancer test (from 2004-2007 and 2008-2010), endoscopy (from 2004-2007 and 2008-2010), and fecal occult blood test (FOBT) (from 2004-2007, 2008-2010, 2011-2016, and 2017-2019). Socioeconomic and demographic characteristics were estimated using the 2000 US Census and linked with county and geographic clusters of screening. These county-level estimates of screening were obtained by extrapolating the Behavioral Risk Factor Surveillance System (BRFSS) and National Health Interview Survey (NHIS) results to the US population using weights for demographic and socioeconomic factors.^25,26,27,28,29^

The BRFSS and NHIS surveys are widely used to estimate national- and state-level prevalences of various health behaviors and preventive services. To obtain data regarding cancer screening use during the study period, we used a set of prespecified definitions. For estimates of mammography, a woman aged 40 years or older must have reported having at least 1 test in her life and should have had the most recent test within the last 2 years by time of the interview. For estimates of Papanicolaou test, a woman aged 21 to 65 years (prior to 2011, the age range was ≥18 years) must have reported having at least 1 test in her life and should have had the most recent test within the last 3 years by the time of interview.

For estimates from 2011 and forward for colonoscopy, within the past 10 years, a person aged 50 to 75 years must have reported having at least 1 test within the past 10 years at the time of interview. For estimates prior to 2011 for colorectal cancer test, a person aged 50 years or older must have reported having at least 1 test (sigmoidoscopy or colonoscopy) in their life or at least 1 home-based FOBT within the past 2 years by the time of interview. For estimates from 2011 and forward for guidance sufficient colorectal cancer screening a person aged 50 to 75 years must have reported having had home-based FOBT within the past year, sigmoidoscopy within the past 5 years, and home-based FOBT within the past 3 years, or colonoscopy within the past 10 years at the time of interview. For estimates from 2011 and forward for colorectal endoscopy, a person aged 50 years or older must have reported having at least 1 test (proctoscopy, sigmoidoscopy, or colonoscopy) in their life. For estimates prior to 2011 for FOBT, a person aged 50 years or older must have reported having at least 1 test using a home test kit in their life and should have had the most recent test within the last 2 years by the time of interview. For estimates from 2011 and forward for FOBT, a person aged 50 to 75 years must have reported having at least 1 FOBT using a home test kit in their life, excluding those who had a colonoscopy within the past 10 years, and they should have had the most recent test within the past year by the time of interview.^24,30,31,32,33,34,35,36^

### Statistical Analysis

To identify geographic clusters of cancer screening within each time period, we performed tests for spatial autocorrelation and cluster detection.^37^ Spatial autocorrelation captures the statistical association and similarity between proximate units in an analysis.^37^ A negative spatial autocorrelation means that cancer screening patterns differ (eg, high screening surrounded by low screening) between adjacent areas, while positive spatial autocorrelation would mean that cancer screening patterns are similar between adjacent areas (eg, high screening surrounded by high screening, low screening surrounded by low screening). The presence of spatial autocorrelation suggests geographic clustering of screening use. These methods are further explained in the eMethods in Supplement 1.

We applied a queen contiguity matrix to define neighboring counties,^37,38^ whereby neighboring counties are defined based on shared borders in any direction. First, we calculated the Global Moran I to determine whether spatial autocorrelation was evident across the contiguous US.^37,38^ If spatial autocorrelation was present, we then calculated the bivariate local indicator of spatial autocorrelation (LISA) to identify the four types of local geographical clusters of county-level cancer screening: (1) persistently high screening counties (high/high); (2) persistently low screening counties (low/low); (3) counties that changed from high to low (high/low); (4) counties that changed from low to high (low/high).^38,39^ For all screening types and temporal comparisons, clustering compared screening prevalence in the earliest period to screening during a later period. *P* values for departures from the null hypothesis (screening is spatially random/no clustering) were obtained from permutation tests.^40^ A significance level of 2-sided *P* ≤ .05 was applied to all statistical tests. All analyses were conducted and maps were developed using R software version 4.3.3. Data were analyzed from 2024 to 2025.

## Results

This study included data from 3142 counties in the US from 1997 to 2019. Specifically, we analyzed 3101 counties for mammography and Papanicolaou test, 3107 counties for colonoscopy, 3100 counties for colorectal cancer test, 3089 counties for endoscopy, and 3090 counties for FOBT. We observed significant variation in sociodemographic characteristics across the 5 cluster types: high/high, low/low, high/low, low/high, and none. In general, counties in low/low and high/low clusters tended to have higher proportions of individuals identifying as Black, lower educational attainment, greater poverty rates, and lower median household incomes and home values compared with counties in high/high clusters or those without significant clustering. These differences were statistically significant across most indicators (analysis of variance: *P* < .001), suggesting consistent associations between structural disadvantage and persistently low screening prevalence. Although these patterns were broadly consistent, the magnitude and direction of disparities fluctuated by screening modality.

### Mammography Screening

Maps of mammography screening in the contiguous US from 1997 to 2019 are displayed in eFigure 1 in Supplement 1. We observed consistent regions of high mammography use in the Northeast and low prevalence in the Southwest of the US over each period, with the highest overall prevalence across the US observed during 2017 to 2019. The Global Moran I coefficient revealed evidence of spatial autocorrelation (eTable 1 in Supplement 1), which attenuated by 83% from 1997 to 1999 (Moran *I* = 0.57) to 2017 to 2019 (Moran *I* = 0.10), suggesting more equal geographic variation in mammography screening in recent years.

**Figure 1.**
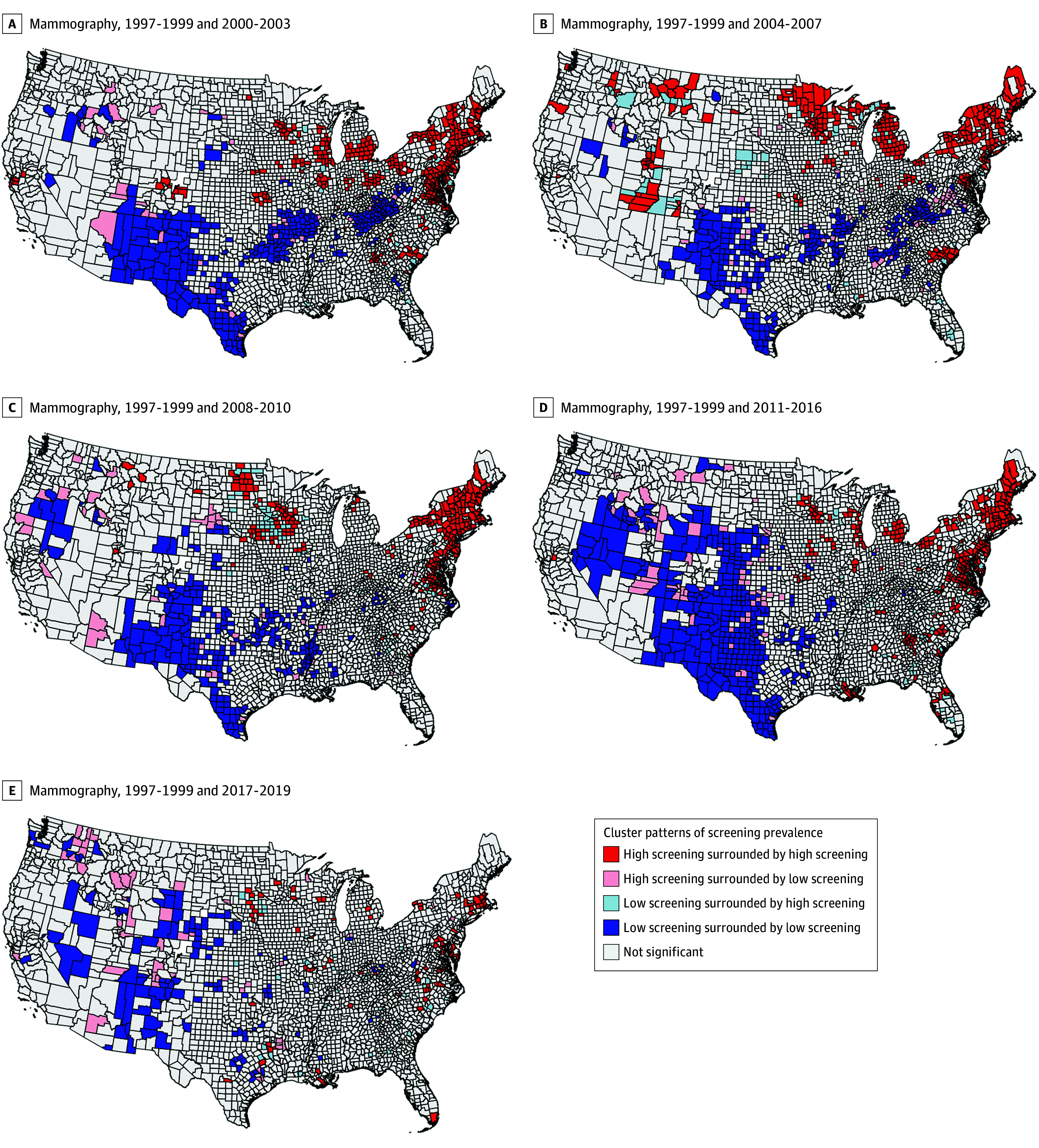
Local Geographical Clusters of County-Level Breast Cancer Screening in the United States from 1997-2019 for Mammography Cluster types based on local mammography screening prevalence and spatial association.

Clusters of bivariate LISA for areas with high and low mammography screening prevalence are displayed in Figure 1 and in the video linked in the eAppendix in Supplement 1. We observed clusters of consistently high (high/high) screening prevalence in the Northeast (Maine, New Hampshire, Vermont, and Massachusetts) over most periods, and consistently low (low/low) clusters in the Southwest (Texas, New Mexico, and Arizona). There were no clear spatial patterns for other cluster types. Sociodemographic characteristics of counties were correlated with cluster types (Table; eTable 2 in Supplement 1). Clusters that changed from low to high prevalence (low/high) had greater disadvantage based on lower socioeconomic status and higher proportion of non-White residents than other cluster types, suggesting some improvement in screening uptake in more disadvantaged areas. For example, county-level median home value was lower among low/high clusters (mean [SD], $74 370.45 [$20 156.66]) compared with high/high clusters (mean [SD], $111 820.41 [$42 759.15]). However, counties classified as low/low also had greater disadvantage relative to high/high, suggesting that in areas characterized by lower socioeconomic status, screening prevalence remained low. This trend also held when comparing county-level median home value in low/high clusters to those in low/low clusters (mean [SD], $75 822.58 [$40 290.14]), although the difference was not as wide.

**Table.** County Demographics by Cluster Patterns and Screening Types for Breast, Cervical, and Colorectal Cancer in the United States

County-level demographic	Cluster pattern, mean (SD)					ANOVA *P* value
	High/high (n = 982)	High/low (n = 358)	Low/high (n = 289)	Low/low (n = 981)	None (n = 15 999)	
**Mammography (1997-1999 vs 2017-2019)**						
No.	92	52	38	118	2801	
Individuals who identify as Black, %	11.83 (15.42)	7.08 (10.49)	9.60 (12.66)	2.75 (6.48)	9.28 (14.85)	<.001
Individuals who identify as White, %	84.15 (15.75)	85.17 (12.85)	87.98 (13.52)	83.80 (16.64)	85.71 (16.02)	.53
<8 y of education and <high school, %	12.21 (4.50)	12.15 (4.25)	13.79 (4.00)	13.45 (3.77)	13.62 (4.71)	.009
Median household income, $	50 951.86 (11 960.91)	41 480.22 (7172.85)	42 509.30 (6713.73)	38 228.32 (7955.15)	41 855.31 (9709.78)	<.001
Below the poverty line, %	10.55 (5.17)	14.32 (4.64)	12.96 (4.16)	16.71 (7.05)	14.23 (6.58)	<.001
Median home value, $	111 820.41 (42 759.15)	91 955.10 (41 589.56)	74 370.45 (20 156.66)	75 822.58 (40 290.14)	83 476.56 (47 849.04)	<.001
**Papanicolaou test (1997-1999 vs 2017-2019)**						
No.	103	39	28	73	2858	
Individuals who identify as Black, %	22.06 (19.41)	2.53 (3.18)	17.00 (20.47)	3.88 (5.50)	8.70 (14.28)	<.001
Individuals who identify as White, %	71.57 (20.34)	91.15 (10.90)	78.95 (20.87)	86.06 (9.35)	86.27 (15.55)	<.001
<8 y of education and <high school, %	13.30 (5.22)	13.58 (3.87)	13.97 (4.82)	14.13 (3.22)	13.55 (4.68)	.79
Median household income, $	52 866.86 (15 178.06)	40 659.30 (7276.67)	50 454.90 (16 540.22)	38 331.47 (5658.88)	41 575.13 (9201.69)	<.001
Below the poverty line, %	11.94 (7.84)	13.78 (5.12)	11.60 (5.17)	15.53 (4.54)	14.27 (6.52)	<.001
Median home value, $	121 050.00 (61 801.96)	75 846.00 (28 126.72)	108 350.00 (63 805.79)	78 113.92 (32 598.99)	82 467.48 (46 299.65)	<.001
**Colonoscopy (2011-2016 vs 2017-2019)**						
No.	209	39	34	212	2613	
Individuals who identify as Black, %	6.68 (11.61)	10.30 (10.55)	7.98 (12.69)	9.94 (15.99)	9.27 (14.85)	.11
Individuals who identify as White, %	90.22 (12.61)	80.32 (12.48)	89.34 (13.10)	75.27 (15.84)	86.13 (15.97)	<.001
<8 y of education and <high school, %	10.51 (3.89)	15.97 (2.82)	11.34 (4.13)	16.93 (3.92)	13.52 (4.62)	<.001
Median household income, $	50 585.32 (11 303.41)	38 717.78 (7421.71)	44 622.64 (6723.62)	12.96 (4.83)	33 637.38 (9335.07)	<.001
Below the poverty line, %	9.61 (3.95)	16.95 (4.81)	11.74 (4.79)	22.28 (7.70)	13.89 (6.10)	<.001
Median home value, $	106 370.14 (41 072.54)	72 531.48 (19 844.13)	81 757.58 (28 131.44)	61 506.73 (34 233.41)	84 340.24 (48 510.20)	<.001
**Colorectal cancer test (2004-2007 vs 2008-2010)**						
No.	257	73	47	245	2478	
Individuals who identify as Black, %	6.14 (10.58)	2.07 (2.86)	5.95 (10.43)	13.38 (21.61)	9.30 (14.29)	<.001
Individuals who identify as White, %	90.17 (12.29)	86.79 (9.21)	90.67 (11.57)	72.68 (19.27)	86.29 (15.55)	<.001
<8 y of education and <high school, %	10.20 (3.38)	13.50 (4.10)	11.32 (3.13)	15.92 (4.50)	13.73 (4.64)	<.001
Median household income, $	52 567.25 (12 064.83)	38 358.39 (9157.19)	50 222.89 (10 125.40)	34 582.38 (7153.39)	41 560.23 (8731.38)	<.001
Below the poverty line, %	9.05 (3.57)	16.08 (5.60)	9.33 (3.76)	21.21 (8.08)	14.08 (6.04)	<.001
Median home value, $	111 995.99 (45 790.69)	67 273.13 (43 866.35)	103 495.56 (38 208.38)	64 082.45 (51 543.65)	83 154.66 (45 910.06)	<.001
**Endoscopy (2004-2007 vs 2008-2010)**						
No.	236	70	49	236	2509	
Individuals who identify as Black, %	7.03 (11.52)	3.63 (7.68)	7.07 (10.69)	9.78 (19.85)	9.47 (14.47)	.002
Individuals who identify as White, %	89.05 (13.07)	87.45 (11.61)	89.32 (12.46)	76.06 (19.81)	86.13 (15.60)	<.001
<8 y of education and <high school, %	10.23 (3.45)	14.66 (4.16)	11.11 (3.26)	15.82 (4.32)	13.69 (4.65)	<.001
Median household income, $	52 939.71 (12 270.18)	36 412.66 (6384.67)	51 993.71 (10 465.03)	33 842.37 (7123.52)	41 669.34 (8623.15)	<.001
Below the poverty line, %	9.05 (3.66)	16.78 (5.47)	8.88 (3.00)	21.51 (8.29)	14.00 (5.94)	<.001
Median home value, $	112 113.62 (47 565.53)	67 848.39 (39 095.70)	109 690.91 (39 473.51)	63 287.45 (50 592.60)	83 233.04 (45 841.54)	<.001
**Fecal occult blood test (2004-2007 vs 2017-2019)**						
No.	85	85	93	97	2740	
Individuals who identify as Black, %	4.05 (9.82)	9.92 (11.24)	6.24 (12.01)	13.13 (13.10)	9.22 (14.92)	<.001
Individuals who identify as White, %	86.80 (17.64)	84.06 (14.45)	89.43 (13.59)	82.15 (13.47)	85.65 (16.06)	.04
<8 y of education and <high school, %	13.63 (3.92)	10.97 (4.15)	15.83 (3.69)	13.11 (4.40)	13.58 (4.70)	<.001
Median household income, $	39 669.56 (7618.57)	52 787.47 (12 153.59)	36 535.81 (9185.34)	48 935.79 (11 696.51)	41 716.71 (9445.45)	<.001
Below the poverty line, %	16.10 (6.56)	10.25 (5.46)	19.03 (8.70)	11.39 (5.14)	14.19 (6.44)	<.001
Median home value, $	94 785.39 (38 978.52)	134 160.51 (111 480.23)	78 006.76 (31 019.64)	105 225.00 (40 192.83)	81 807.25 (43 996.46)	<.001

Abbreviation: ANOVA, analysis of variance.

### Papanicolaou Test Screening

Maps of Papanicolaou test screening in the contiguous US from 1997 to 2019 are displayed in eFigure 2 in Supplement 1. We observed consistent regions of high prevalence of Papanicolaou test screening in the East and Midwest and lower prevalence in the West in the earlier years (1997 to 2003), with most of the US achieving high prevalence of Papanicolaou test screening rates by 2019. The Global Moran I coefficient revealed evidence of spatial autocorrelation (eTable 1 in Supplement 1), which attenuated by 85% from 1997 to 1999 (Moran *I* = 0.44) to 2017 to 2019 (Moran *I* = 0.07), suggesting more equal geographic variation in Papanicolaou test screening in recent years.

**Figure 2.**
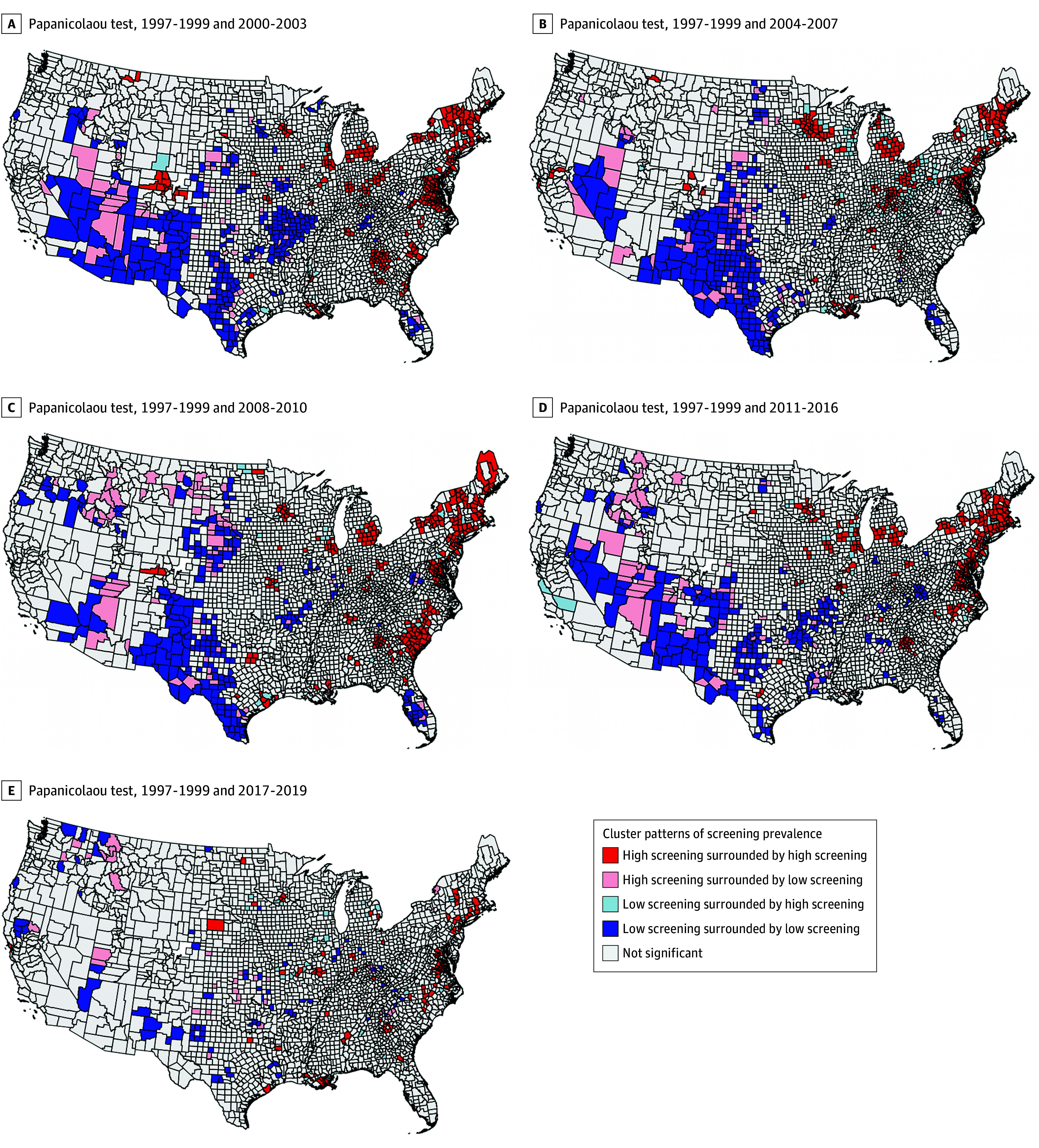
Local Geographical Clusters of County-Level Cervical Cancer Screening in the United States from 1997-2019 for Papanicolaou test Cluster types based on local Papanicolaou test screening prevalence and spatial association.

Clusters of bivariate LISA for areas with high and low Papanicolaou test screening prevalence are displayed in Figure 2 and in the video linked in the eAppendix in Supplement 1. We observed clusters of high/high screening prevalence in the general East (from the Northeast to the South through both North Carolina and South Carolina, until Georgia) over most periods, and consistent low/low clusters in the Southwest (Texas, New Mexico, and Arizona). No other spatial patterns emerged. Once again, we found that sociodemographic characteristics of counties were correlated with cluster types (Table; eTable 3 in Supplement 1). Clusters of low/high had greater disadvantage based on lower socioeconomic status and higher proportion of non-White residents than other cluster types, suggesting some improvement in screening uptake in more disadvantaged areas. For example, county-level median home value was lower among those in low/high clusters (mean [SD], $108 350.00 [$63 805.79]) compared to those in high/high clusters (mean [SD], $121 050.00 [$61 801.96]). As with mammography screening results, compared with high/high clusters, counties with low/low cluster type also had greater socioeconomic disadvantage. However, unlike mammography screening results, low/high clusters did not have greater disadvantage compared with low/low screening.

### Colorectal Cancer Screening

Multiple modalities were individually assessed to study colorectal cancer screening use. Maps of colonoscopy screening from 2011 to 2019, colorectal cancer test screening from 2004 to 2010, and endoscopy screening from 2004 to 2010 are presented in in eFigure 3 in Supplement 1. Maps of FOBT screening from 2004 to 2019 are presented in eFigure 4 in Supplement 1. By 2010, most counties had achieved screening rates greater than 50% for colonoscopy, colorectal cancer test, and endoscopy, with higher rates in the East and Midwest. In contrast, there was a decrease in FOBT screening with time. The Global Moran I coefficient revealed evidence of spatial autocorrelation (eTable 1 in Supplement 1), which attenuated 23.4% from 2011 to 2016 to 2017 to 2019 for colonoscopy, 12.3% from 2004 to 2007 to 2008 to 2010 for colorectal cancer test, and 14.0% from 2004 to 2007 to 2008 to 2010 for endoscopy. Similar trends did not hold for FOBT. These finding are consistent with observations of higher prevalence of screening for most tests, suggesting there was less heterogeneity in the screening prevalence estimates and increased saturation with diffusion of screening usage.

**Figure 3.**
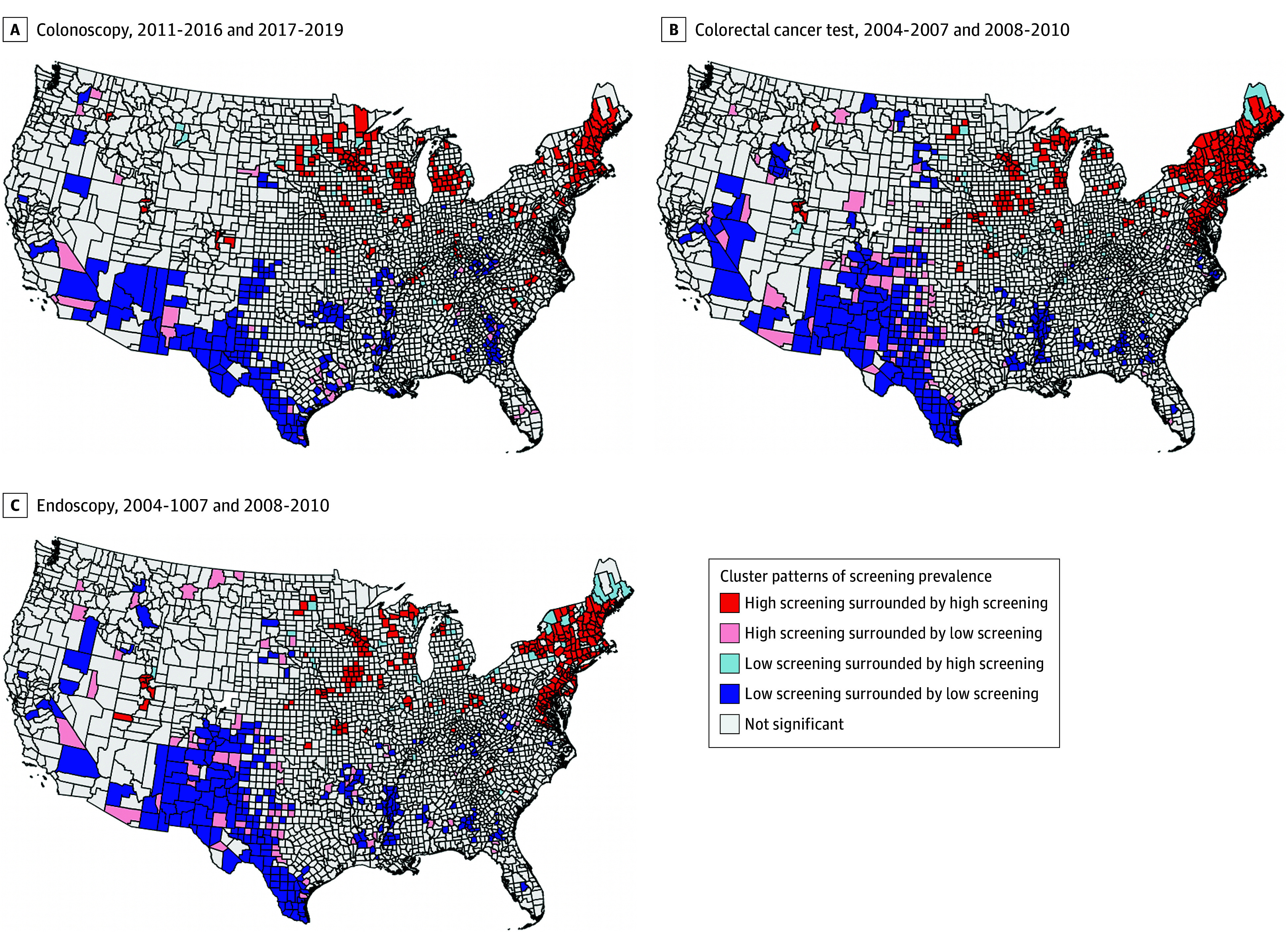
Local Geographical Clusters of County-Level Colorectal Cancer Screening in the United States from 2004-2019 for Colonoscopy, Colorectal Cancer Test, and Endoscopy Cluster types based on local colonoscopy, colorectal cancer test, and endoscopy screening prevalence and spatial association.

**Figure 4.**
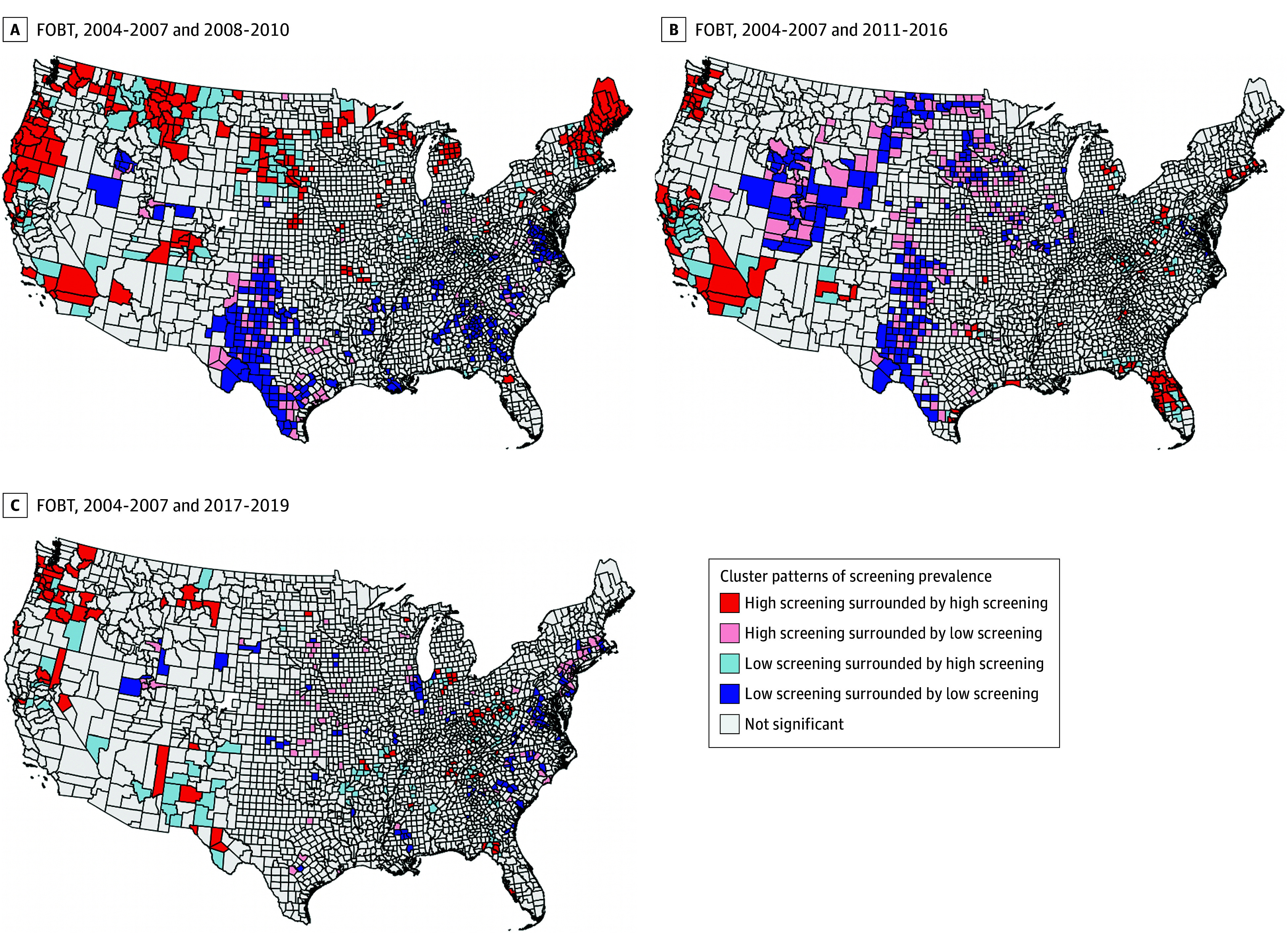
Local Geographical Clusters of County-Level Colorectal Cancer Screening in the United States from 2004-2019 for Fecal Occult Blood Test Cluster types based on local fecal occult blood test screening prevalence and spatial association.

Clusters of bivariate LISA for areas with high and low screening prevalence are displayed by modality in Figure 3 and Figure 4. Overall, we observed clusters of high/high screening prevalence in the Northeast (Maine, New Hampshire, Vermont, and Massachusetts) over most periods, and consistent low/low clusters in the Southwest (Texas, New Mexico, and Arizona) for colonoscopy, colorectal cancer test, and endoscopy (Figure 3). There were no clear spatial patterns for FOBT (Figure 4). Clusters of low/high had greater disadvantage based on lower socioeconomic status and higher proportion of non-White residents than other cluster types, suggesting some improvement in screening uptake in more disadvantaged areas. For example, county-level median home value and county-level median household income were consistently lower among those in low/high clusters compared to those in high/high clusters for all 4 screening modalities (Table; eTables 4-7 in Supplement 1). However, similar to the other cancer screening types, greater socioeconomic disadvantage was also higher in counties classified as low/low compared with those classified as high/high. Additionally, counties classified as low/high had less socioeconomic disadvantage compared with those classified as low/low.

## Discussion

In this cross-sectional study of geographic clusters of screening use in the US, we observed that global spatial autocorrelation declined over time, suggesting increasing screening in later years reduced geographic disparities. Clusters of persistently high screening were observed in the Northeast. Although secular increases in screening occurred in all parts of the US, the relative clustering of high screening remained stable. Clusters of persistently low screening in the Southwest were also observed. The use of mammography, Papanicolaou test, colonoscopy, colorectal cancer test, and endoscopy increased over the study period but decreased for FOBT. This could be explained by the rise in the use of endoscopy or colorectal cancer test screening methods compared with FOBT to screen for colorectal cancer.^41^

Transportation barriers can affect access to preventive health care. A 2023 study found that non-Hispanic Black individuals had 3.54 times higher odds of relying on public transit compared with non-Hispanic White individuals, and this was inversely proportional to their income level.^42^ Non-Hispanic Black individuals with lower incomes ($25 000-$49 999) faced an additional 81.9 minutes of travel time due to reliance on public transportation, compared with 25.5 minutes for their non-Hispanic White counterparts.^42^ Another influence on screening is the relative lack of facilities in rural compared with urban communities in the US. In 2017, the average US resident spent 27.1 minutes in transit to receive medical care; urban residents spend 25.5 minutes and rural residents spent 34.2 minutes.^43^ In 2020, there were 8.0 primary care physicians per 10 000 urban area residents, compared with 5.1 physicians per 10 000 rural residents.^44^ There were 134 specialists per 10 000 residents in urban areas compared with 40 specialists per 10 000 rural residents.^26,27^ It is evident that rural residents face a higher travel burden to access both primary care and specialty care.^45^ These observations may explain why some communities with low cancer screening tend to be surrounded by other communities with low cancer screening.

Rural residents may have lower educational attainment, lower incomes, and lower insurance coverage, which may further exacerbate access to cancer screening.^46^ This aligns with previous literature, which suggests that access to health care facilities, limited infrastructure, fewer primary care practices, lack of technological interventions, and lower socioeconomic status may serve as barriers and partially explain the variations in cancer screening patterns among regions.^46,47,48,49^ However, this pattern is also observed in urban residents with lower education and income levels: women who are less educated or from a lower socioeconomic group were less likely to obtain routine mammogram or Papanicolaou test screening compared with women who were more educated or from a higher socioeconomic group, even if they resided in the same community.^50,51,52,53,54^

These socioeconomic and demographic factors hinder the diffusion of cancer screening among communities. These observations are consistent with our study that found that areas with lower education and income levels tended to have lower screening rates. Although counties that started with relatively low screening had similar sociodemographic characteristics, some experienced increases and had relatively higher screening in the later time period. Further examination of access barriers and knowledge among persistently low screening areas vs those that changed could inform outreach efforts.

### Strengths and Limitations

The major strength of this study is that it is the largest study to our knowledge to empirically evaluate spatial and temporal patterns of screening use over a 23-year period. Past studies have rarely evaluated geographic and temporal changes in screening. Among those studies, most have analyzed only a single state or a single screening modality. Our study examined patterns across the contiguous US for 6 different screening modalities. We assessed local and global clusters throughout various periods of time, starting at the earliest date at which data were available from BRFSS and NHIS. Our study design allowed for a thorough examination of the diffusion of cancer screening during a time when the appropriateness of multiple cancer screening guidelines was debated.^55,56^

Our study has a few limitations. Our county-level estimates of screening were modeled using BRFSS, NHIS, and the US Census. While BRFSS and NHIS are powered for state and national estimates, modeled estimates may be susceptible to sampling biases. The small area estimates are based on underlying model assumptions that take into consideration the demographic profile of each geographic area.^57^ To reduce variance and address this limitation, BRFSS and NHIS have corrected for nonresponse and noncoverage biases with their model assumptions and covariate selection.^57^ We did not have data on specific health systems characteristics (eg, clinician preference, facilities where screening is performed, insurance) that may directly drive changes in prevalence. We were also restricted to using screening time intervals that were available from the SAE and NCI, instead of those according to USPSTF guidelines. For example, although the USPSTF recommends Papanicolaou test and human papillomavirus testing every 5 years for individuals aged 30 to 65 years, we had to use the 3-year Papanicolaou test screening time interval, which could potentially underestimate the actual up-to-date screening rates.^2,3,4^ Furthermore, although the spatial cluster detection method is sensitive to the number and arrangement of neighboring counties—which can vary significantly due to differences in county size and shape across the US—this does not fully explain the patterns we observed. Geographic variation in practice patterns and availability of clinicians and changes in screening recommendations are more likely to explain geographic variation in screening.^58,59,60,61^

## Conclusions

This cross-sectional study found that despite secular increases that reduced geographic variation in screening, local clusters of high and low screening persisted in the Northeast and Southwest US, respectively. Future studies could incorporate health care access characteristics to explain why areas of low screening did not catch up to optimize cancer screening practices.

## Notes

Supplement 1.
eMethods.
**eTable 1.** Moran's I Statistical Values and P-Values by Screening Type and Year **eTable 2.** County Demographics by Cluster Patterns for Mammography in the United States From 1997-1999 vs. 2017-2019. **eTable 3.** County Demographics by Cluster Patterns for Pap Smear in the United States From 1997-1999 vs. 2017-2019 **eTable 4.** County Demographics by Cluster Patterns for Colonoscopy in the United States From 2011-2016 vs. 2017-2019 **eTable 5.** County Demographics by Cluster Patterns for Colorectal Cancer Test in the United States From 2004-2007 vs. 2008-2010 **eTable 6.** County Demographics by Cluster Patterns for Endoscopy in the United States From 2004-2007 vs. 2008-2010 **eTable 7.** County Demographics by Cluster Patterns for Fecal Occult Blood Test in the United States from 2004-2007 vs. 2017-2019 **eFigure 1.** Prevalence of Breast Cancer Screening in the United States from 1997-2019 for Mammography **eFigure 2.** Prevalence of Cervical Cancer Screening in the United States from 1997-2019 for Pap Smear **eFigure 3.** Prevalence of Colorectal Cancer Screening in the United States from 2004-2019 for Colonoscopy, Colorectal Cancer Test, and Endoscopy **eFigure 4.** Prevalence of Colorectal Cancer Screening in the United States from 2004-2019 for Fecal Occult Blood Test **eAppendix.** Local Geographical Clusters of County-Level Cancer Screenings in the US

Supplement 2.
Data Sharing Statement
