# Supplementary material for: Geographic and Temporal Patterns of Screening for Breast, Cervical, and Colorectal Cancer in the US, 1997-2019
Source: JAMA Netw Open. 2025 Oct 17;8(10):e2537905. doi: 10.1001/jamanetworkopen.2025.37905 (PMC12534853; doi:10.1001/jamanetworkopen.2025.37905)
Supplement: Supplement 1. — eMethods. eTable 1. Moran's I Statistical Values and P-Values by Screening Type and Year eTable 2. County Demographics by Cluster Patterns for Mammography in the United States From 1997-1999 vs. 2017-2019. eTable 3. County Demographics by Cluster Patterns for Pap Smear in the United States From 1997-1999 vs. 2017-2019 eTable 4. County Demographics by Cluster Patterns for Colonoscopy in the United States From 2011-2016 vs. 2017-2019 eTable 5. County Demographics by Cluster Patterns for Colorectal Cancer Test in the United States From 2004-2007 vs. 2008-2010 eTable 6. County Demographics by Cluster Patterns for Endoscopy in the United States From 2004-2007 vs. 2008-2010 eTable 7. County Demographics by Cluster Patterns for Fecal Occult Blood Test in the United States from 2004-2007 vs. 2017-2019 eFigure 1. Prevalence of Breast Cancer Screening in the United States from 1997-2019 for Mammography eFigure 2. Prevalence of Cervical Cancer Screening in the United States from 1997-2019 for Pap Smear eFigure 3. Prevalence of Colorectal Cancer Screening in the United States from 2004-2019 for Colonoscopy, Colorectal Cancer Test, and Endoscopy eFigure 4. Prevalence of Colorectal Cancer Screening in the United States from 2004-2019 for Fecal Occult Blood Test eAppendix. Local Geographical Clusters of County-Level Cancer Screenings in the US [file jamanetwopen-e2537905-s001.pdf]

Supplemental Online Content

Pradhan P, Iyer HS, Rebbeck TR. Geographic and temporal patterns of screening for breast, cervical, and colorectal cancer in the US, 1997-2019. *JAMA Netw Open*. 2025;8(10):e2537905. doi:10.1001/jamanetworkopen.2025.37905

eMethods.
eTable 1. Moran's I Statistical Values and P-Values by Screening Type and Year
eTable 2. County Demographics by Cluster Patterns for Mammography in the United States From 1997-1999 vs. 2017-2019.
eTable 3. County Demographics by Cluster Patterns for Pap Smear in the United States From 1997-1999 vs. 2017-2019
eTable 4. County Demographics by Cluster Patterns for Colonoscopy in the United States From 2011-2016 vs. 2017-2019
eTable 5. County Demographics by Cluster Patterns for Colorectal Cancer Test in the United States From 2004-2007 vs. 2008-2010
eTable 6. County Demographics by Cluster Patterns for Endoscopy in the United States From 2004-2007 vs. 2008-2010
eTable 7. County Demographics by Cluster Patterns for Fecal Occult Blood Test in the United States from 2004-2007 vs. 2017-2019
eFigure 1. Prevalence of Breast Cancer Screening in the United States from 1997-2019 for Mammography
eFigure 2. Prevalence of Cervical Cancer Screening in the United States from 1997-2019 for Pap Smear
eFigure 3. Prevalence of Colorectal Cancer Screening in the United States from 2004-2019 for Colonoscopy, Colorectal Cancer Test, and Endoscopy
eFigure 4. Prevalence of Colorectal Cancer Screening in the United States from 2004-2019 for Fecal Occult Blood Test
eAppendix. Local Geographical Clusters of County-Level Cancer Screenings in the US

This supplemental material has been provided by the authors to give readers additional information about their work.

Supplemental Methods for Local Indicator of Spatial Autocorrelation

The bivariate local indicator of spatial autocorrelation applies a “weight matrix” that defines neighbors for each county. We provide a general formula used to describe local indicator of spatial autocorrelation measures[1]:

$$I_i = \frac{1}{\sum_i z_i^2} * z_i \sum_j w_{ij} z_j$$

Where *I* represents the local statistical value (in this case, screening prevalence) in county indexed by i, j is an index for the number of neighboring counties for county *I, z* is the value of screening prevalence, and w_{ij} is the spatial weights matrix used to define neighbors for each county, with number of rows and columns equal to the total number of counties, indexed by *i* and j.

Less formally, the local indicator of spatial autocorrelation is the product of the screening prevalence in county *i* with the “spatial lag” (the weighted sum of screening prevalence for all neighbors).

The method is sensitive to the arrangement of polygons and definition of the weights matrix. While polygons with fewer neighbors do contribute to the calculation of the Local Indicator of Spatial Autocorrelation, neighboring values will contribute more relative weight to the calculation than polygons with more neighbors.

References:

1. Anselin L. Exploring Spatial Data with GeoDaTM : A Workbook.

eTable 1. Moran's I Statistical Values and P-Values by Screening Type and Year							
Screening	Year	Moran's I	Expectation	Variance	Standard Deviate	P-Value	Correlation
Mammography	1997-1999	0.57	-0.00032	0.00012	52.63	<0.001	Positive
	2000-2003	0.51	-0.00032	0.00012	47.36	<0.001	Positive
	2004-2007	0.36	-0.00033	0.00012	33.29	<0.001	Positive
	2008-2010	0.32	-0.00032	0.00012	29.89	<0.001	Positive
	2011-2016	0.52	-0.00032	0.00012	46.68	<0.001	Positive
	2017-2019	0.10	-0.00032	0.00012	9.18	<0.001	Positive
Pap Smear	1997-1999	0.44	-0.00032	0.00012	40.87	<0.001	Positive
	2000-2003	0.45	-0.00032	0.00012	41.82	<0.001	Positive
	2004-2007	0.42	-0.00033	0.00012	39.07	<0.001	Positive
	2008-2010	0.34	-0.00032	0.00012	31.80	<0.001	Positive
	2011-2016	0.33	-0.00032	0.00012	30.92	<0.001	Positive
	2017-2019	0.07	-0.00032	0.00012	6.08	<0.001	Positive
Colonoscopy	2011-2016	0.39	-0.00032	0.00012	35.67	<0.0001	Positive
	2017-2019	0.29	-0.00032	0.00012	27.34	<0.001	Positive
Colorectal Cancer Test	2004-2007	0.41	-0.00032	0.00012	38.01	<0.001	Positive
	2008-2010	0.36	-0.00032	0.00012	33.33	<0.001	Positive
Endoscopy	2004-2007	0.41	-0.00032	0.00012	37.60	<0.001	Positive
	2008-2010	0.35	-0.00032	0.00012	32.32	<0.001	Positive
FOBT	2004-2007	0.29	-0.00033	0.00012	27.17	<0.001	Positive
	2008-2010	0.33	-0.00032	0.00012	30.81	<0.001	Positive
	2011-2016	0.32	-0.00032	0.00012	30.34	<0.001	Positive
	2017-2019	0.13	-0.00032	0.00012	12.05	<0.001	Positive

eTable 2: County Demographics by Cluster Patterns for Mammography in the United States from 1997-1999 vs. 2017-2019.						
**County Demographic (Mean ± SD)**	**Cluster Pattern (N=Number of Counties)**					***Anova***
	**High/High (N=92)**	**High/Low (N=52)**	**Low/High (N=38)**	**Low/Low (N=118)**	**None (N=2,801)**	
**Population**						
Total in the county (N)	179,620.92 (249,896.45)	32,854.12 (59,764.31)	38,984.95 (48,984.27)	26,896.65 (45,861.80)	90,703.86 (303,358.84)	0.002
Children (< 18) (%)	24.62 (2.39)	25.72 (2.94)	25.04 (2.18)	26.86 (4.49)	25.46 (3.17)	<0.001
**Race**						
Identifies as Black (%)	11.83 (15.42)	7.08 (10.49)	9.60 (12.66)	2.75 (6.48)	9.28 (14.85)	<0.001
Identifies as White (%)	84.15 (15.75)	85.17 (12.85)	87.98 (13.52)	83.80 (16.64)	85.71 (16.02)	0.53
Foreign-born (%)	4.59 (4.05)	3.56 (3.69)	2.21 (3.00)	3.87 (5.40)	3.40 (4.88)	0.05
**Education for Adults (25+ years)**						
At least 8 years of education (%)	7.72 (4.50)	7.10 (4.17)	9.49 (3.11)	9.59 (5.74)	9.17 (5.29)	0.004
< 8 years of education and < high school (%)	12.21 (4.50)	12.15 (4.25)	13.79 (4.00)	13.45 (3.77)	13.62 (4.71)	0.009
Completed high school (%)	31.66 (5.96)	33.02 (5.90)	37.15 (5.75)	32.86 (5.77)	34.86 (6.59)	<0.001
Bachelor’s degree or higher (%)	22.54 (9.85)	18.67 (7.70)	14.08 (5.45)	16.00 (6.83)	16.31 (7.69)	<0.001
**Income (N or %)**						
Median household income ($)	50,951.86 (11,960.91)	41,480.22 (7,172.85)	42,509.30 (6,713.73)	38,228.32 (7,955.15)	41,855.31 (9,709.78)	<0.001
Below the poverty line (%)	10.55 (5.17)	14.32 (4.64)	12.96 (4.16)	16.71 (7.05)	14.23 (6.58)	<0.001
Unemployment rate (%)	2.84 (1.33)	3.24 (1.56)	2.98 (0.90)	4.82 (3.08)	3.37 (1.86)	<0.001

eTable 2: County Demographics by Cluster Patterns for Mammography in the United States from 1997-1999 vs. 2017-2019.						
**County Demographic (Mean ± SD)**	**Cluster Pattern (N=Number of Counties)**					***Anova***
	**High/High (N=92)**	**High/Low (N=52)**	**Low/High (N=38)**	**Low/Low (N=118)**	**None (N=2,801)**	
Male unemployment rate (%)	3.22 (1.16)	4.76 (2.49)	3.79 (1.17)	4.03 (2.13)	4.03 (2.13)	<0.001
**Home Values**						
Median home value ($)	111,820.41 (42,759.15)	91,955.10 (41,589.56)	74,370.45 (20,156.66)	75,822.58 (40,290.14)	83,476.56 (47,849.04)	<0.001
Median rent ($)	540.59 (132.09)	443.63 (96.61)	409.70 (63.52)	409.10 (92.98)	438.68 (122.17)	<0.001
Occupied housing units (%)	90.91 (7.28)	78.56 (15.02)	89.25 (5.01)	81.50 (11.05)	85.90 (9.36)	<0.001
Female-headed households with children but no husband (%)	21.95 (8.42)	19.92 (5.07)	20.78 (6.39)	20.18 (6.16)	20.77 (7.48)	0.45
**Welfare**						
Households receiving some form of income assistance (%)	38.08 (9.05)	34.80 (7.93)	33.71 (7.25)	30.02 (8.37)	33.41 (9.65)	<0.001
Households receiving welfare assistance (%)	7.07 (3.33)	7.85 (3.08)	7.95 (2.36)	10.66 (5.91)	8.48 (4.21)	<0.001
**Index Measurements**						
Income ICE index	0.0954 (0.1871)	-0.0623 (0.1329)	-0.0443 (0.1239)	-0.1164 (0.1496)	-0.0540 (0.1723)	<0.001
Race ICE index	0.7603 (0.2859)	0.8042 (0.2009)	0.8070 (0.2377)	0.8307 (0.1743)	0.7937 (0.2738)	0.49
Race and income ICE index	0.2508 (0.1409)	0.1730 (0.0865)	0.1769 (0.1026)	0.1697 (0.0817)	0.1728 (0.1260)	<0.001

eTable 3: County Demographics by Cluster Patterns for Pap Smear in the United States from 1997-1999 vs. 2017-2019.						
**County Demographic (Mean ± SD)**	**Cluster Pattern (N=Number of Counties)**					***Anova***
	**High/High (N=103)**	**High/Low (N=39)**	**Low/High (N=28)**	**Low/Low (N=73)**	**None (N=2,858)**	
**Population**						
Total in the county (N)	227,200.92 (410,855.46)	37,821.52 (55,071.61)	156,364.40 (182,287.43)	57,288.18 (155,823.56)	84,458.14 (290,306.67)	<0.001
Children (< 18) (%)	25.82 (3.97)	25.46 (3.17)	25.16 (1.05)	25.79 (2.94)	25.44 (3.16)	0.60
**Race**						
Identifies as Black (%)	22.06 (19.41)	2.53 (3.18)	17.00 (20.47)	3.88 (5.50)	8.70 (14.28)	<0.001
Identifies as White (%)	71.57 (20.34)	91.15 (10.90)	78.95 (20.87)	86.06 (9.35)	86.27 (15.55)	<0.001
Foreign-born (%)	5.73 (6.00)	1.99 (2.65)	6.32 (7.78)	5.47 (5.53)	3.28 (4.72)	<0.001
**Education for Adults (25+ years)**						
At least 8 years of education (%)	7.56 (3.93)	8.42 (4.32)	7.40 (3.10)	9.96 (4.38)	9.18 (5.34)	0.002
< 8 years of education and < high school (%)	13.30 (5.22)	13.58 (3.87)	13.97 (4.82)	14.13 (3.22)	13.55 (4.68)	0.79
Completed high school (%)	30.59 (6.91)	36.14 (5.36)	33.88 (4.51)	33.02 (5.86)	34.94 (6.51)	<0.001
Bachelor’s degree or higher (%)	22.94 (12.33)	15.89 (5.67)	19.43 (10.14)	15.02 (4.70)	16.22 (7.44)	<0.001
**Income (N or %)**						
Median household income ($)	52,866.86 (15,178.06)	40,659.30 (7,276.67)	50,454.90 (16,540.22)	38,331.47 (5,658.88)	41,575.13 (9,201.69)	<0.001
Below the poverty line (%)	11.94 (7.84)	13.78 (5.12)	11.60 (5.17)	15.53 (4.54)	14.27 (6.52)	<0.001
Unemployment rate (%)	3.09 (2.31)	4.04 (2.98)	3.29 (1.62)	4.05 (1.64)	3.37 (1.85)	<0.001

eTable 3: County Demographics by Cluster Patterns for Pap Smear in the United States from 1997-1999 vs. 2017-2019.						
**County Demographic (Mean ± SD)**	**Cluster Pattern (N=Number of Counties)**					***Anova***
	**High/High (N=103)**	**High/Low (N=39)**	**Low/High (N=28)**	**Low/Low (N=73)**	**None (N=2,858)**	
Male unemployment rate (%)	3.79 (2.98)	3.55 (1.86)	4.05 (1.09)	3.87 (1.50)	3.73 (1.66)	0.82
**Home Values**						
Median home value ($)	121,050.00 (61,801.96)	75,846.00 (28,126.72)	108,350.00 (63,805.79)	78,113.92 (32,598.99)	82,467.48 (46,299.65)	<0.001
Median rent ($)	579.08 (168.93)	409.82 (82.70)	531.60 (155.20)	433.16 (82.91)	433.95 (116.17)	<0.001
Occupied housing units (%)	90.55 (6.78)	86.00 (6.42)	89.98 (6.61)	84.36 (9.68)	85.64 (9.65)	<0.001
Female-headed households with children but no husband (%)	24.68 (10.02)	18.91 (4.26)	23.98 (8.81)	19.73 (4.06)	20.62 (7.32)	<0.001
**Welfare**						
Households receiving some form of income assistance (%)	35.09 (11.01)	32.14 (7.35)	36.92 (11.66)	31.46 (6.09)	33.47 (9.61)	0.04
Households receiving welfare assistance (%)	7.52 (4.43)	9.37 (4.72)	7.87 (3.67)	9.37 (3.07)	8.51 (4.23)	0.01
**Index Measurements**						
Income ICE index	0.1267 (0.2230)	-0.0782 (0.1427)	-0.0640 (0.2326)	-0.1045 (0.1103)	-0.0588 (0.1668)	<0.001
Race ICE index	0.5440 (0.3543)	0.8973 (0.1118)	0.6563 (0.3871)	0.8434 (0.1136)	0.8043 (0.2632)	<0.001
Race and income ICE index	0.2174 (0.1946)	0.1912 (0.0751)	0.2057 (0.1919)	0.1682 (0.0587)	0.1728 (0.1221)	<0.001

eTable 4: County Demographics by Cluster Patterns for Colonoscopy in the United States from 2011-2016 vs. 2017-2019.						
**County Demographic (Mean ± SD)**	**Cluster Pattern (N=Number of Counties)**					***Anova***
	**High/High (N=209)**	**High/Low (N=39)**	**Low/High (N=34)**	**Low/Low (N=212)**	**None (N=2,613)**	
**Population**						
Total in the county (N)	143,605.51 (214,911.66)	137,734.19 (469,371.75)	65,582.45 (92,680.86)	85,851.37 (284,929.14)	85,023.68 (294,863.60)	0.04
Children (< 18) (%)	24.89 (2.46)	24.70 (3.74)	24.62 (2.60)	27.90 (4.06)	25.35 (3.08)	<0.001
**Race**						
Identifies as Black (%)	6.68 (11.61)	10.30 (10.55)	7.98 (12.69)	9.94 (15.99)	9.27 (14.85)	0.11
Identifies as White (%)	90.22 (12.61)	80.32 (12.48)	89.34 (13.10)	75.27 (15.84)	86.13 (15.97)	<0.001
Foreign-born (%)	3.50 (3.30)	6.04 (5.07)	2.81 (3.00)	7.40 (8.59)	3.07 (4.36)	<0.001
**Education for Adults (25+ years)**						
At least 8 years of education (%)	6.36 (3.37)	11.01 (3.72)	6.94 (2.39)	16.30 (7.84)	8.76 (4.67)	<0.001
< 8 years of education and < high school (%)	10.51 (3.89)	15.97 (2.82)	11.34 (4.13)	16.93 (3.92)	13.52 (4.62)	<0.001
Completed high school (%)	33.97 (6.75)	33.72 (5.74)	36.81 (6.57)	30.55 (5.59)	35.10 (6.51)	<0.001
Bachelor’s degree or higher (%)	21.58 (9.59)	14.55 (6.47)	17.87 (8.50)	12.96 (4.83)	16.38 (7.63)	<0.001
**Income (N or %)**						
Median household income ($)	50,585.32 (11,303.41)	38,717.78 (7,421.71)	44,622.64 (6,723.62)	12.96 (4.83)	33,637.38 (9,335.07)	<0.001
Below the poverty line (%)	9.61 (3.95)	16.95 (4.81)	11.74 (4.79)	22.28 (7.70)	13.89 (6.10)	<0.001
Unemployment rate (%)	2.83 (1.32)	3.61 (1.57)	2.90 (1.01)	5.43 (2.87)	3.27 (1.75)	<0.001

eTable 4: County Demographics by Cluster Patterns for Colonoscopy in the United States from 2011-2016 vs. 2017-2019.						
**County Demographic (Mean ± SD)**	**Cluster Pattern (N=Number of Counties)**					***Anova***
	**High/High (N=209)**	**High/Low (N=39)**	**Low/High (N=34)**	**Low/Low (N=212)**	**None (N=2,613)**	
Male unemployment rate (%)	3.52 (1.12)	3.80 (1.56)	3.69 (1.18)	4.52 (2.09)	3.69 (1.75)	<0.001
**Home Values**						
Median home value ($)	106,370.14 (41,072.54)	72,531.48 (19,844.13)	81,757.58 (28,131.44)	61,506.73 (34,233.41)	84,340.24 (48,510.20)	<0.001
Median rent ($)	511.77 (121.06)	442.85 (98.80)	434.64 (97.59)	382.01 (95.71)	439.35 (122.03)	<0.001
Occupied housing units (%)	88.03 (10.07)	81.87 (8.06)	88.07 (11.26)	82.50 (9.13)	86.00 (9.45)	<0.001
Female-headed households with children but no husband (%)	19.61 (6.71)	21.90 (5.28)	19.40 (6.54)	23.60 (7.01)	20.64 (7.51)	<0.001
**Welfare**						
Households receiving some form of income assistance (%)	40.76 (7.83)	29.87 (7.35)	38.71 (8.56)	23.50 (7.21)	33.67 (9.26)	<0.001
Households receiving welfare assistance (%)	6.63 (2.51)	9.06 (3.00)	6.91 (2.60)	12.95 (6.05)	8.30 (3.97)	<0.001
**Index Measurements**						
Income ICE index	0.0899 (0.1750)	-0.1045 (0.1452)	-0.0168 (0.1110)	-0.1855 (0.1441)	-0.0517 (0.1672)	<0.001
Race ICE index	0.8591 (0.2212)	0.7366 (0.1959)	0.8401 (0.2369)	0.6966 (0.2555)	0.7970 (0.2748)	<0.001
Race and income ICE index	0.2665 (0.1221)	0.1416 (0.0793)	0.1944 (0.0854)	0.1071 (0.1066)	0.1733 (0.1237)	<0.001

eTable 5: County Demographics by Cluster Patterns for Colorectal Cancer Test in the United States from 2004-2007 vs. 2008-2010.						
**County Demographic (Mean ± SD)**	**Cluster Pattern (N=Number of Counties)**					***Anova***
	**High/High (N=257)**	**High/Low (N=73)**	**Low/High (N=47)**	**Low/Low (N=245)**	**None (N=2,478)**	
**Population**						
Total in the county (N)	182,693.45 (266,121.90)	22,357.33 (50,255.10)	134,631.00 (162,270.90)	47,958.66 (143,465.10)	84,865.94 (310,570.30)	<0.001
Children (< 18) (%)	25.01 (2.46)	26.27 (3.48)	24.89 (2.12)	27.91 (4.16)	25.26 (3.05)	<0.001
**Race**						
Identifies as Black (%)	6.14 (10.58)	2.07 (2.86)	5.95 (10.43)	13.38 (21.61)	9.30 (14.29)	<0.001
Identifies as White (%)	90.17 (12.29)	86.79 (9.21)	90.67 (11.57)	72.68 (19.27)	86.29 (15.55)	<0.001
Foreign-born (%)	4.53 (4.91)	5.00 (4.51)	3.34 (2.80)	6.29 (7.18)	3.00 (4.44)	<0.001
**Education for Adults (25+ years)**						
At least 8 years of education (%)	5.39 (2.18)	10.43 (6.12)	6.32 (2.81)	14.09 (7.27)	9.04 (4.85)	<0.001
< 8 years of education and < high school (%)	10.20 (3.38)	13.50 (4.10)	11.32 (3.13)	15.92 (4.50)	13.73 (4.64)	<0.001
Completed high school (%)	33.66 (7.10)	32.07 (5.34)	36.27 (7.26)	30.80 (5.50)	35.25 (6.45)	<0.001
Bachelor's degree or higher (%)	23.77 (9.84)	16.53 (8.08)	19.86 (8.67)	14.40 (6.14)	15.85 (7.21)	<0.001
**Income (N or %)**						
Median household income ($)	52,567.25 (12,064.83)	38,358.39 (9,157.19)	50,222.89 (10,125.40)	34,582.38 (7,153.39)	41,560.23 (8,731.38)	<0.001
Below the poverty line (%)	9.05 (3.57)	16.08 (5.60)	9.33 (3.76)	21.21 (8.08)	14.08 (6.04)	<0.001
Unemployment rate (%)	2.79 (1.31)	3.80 (1.84)	2.78 (1.48)	4.69 (2.84)	3.33 (1.78)	<0.001

eTable 5: County Demographics by Cluster Patterns for Colorectal Cancer Test in the United States from 2004-2007 vs. 2008-2010.						
**County Demographic (Mean ± SD)**	**Cluster Pattern (N=Number of Counties)**					***Anova***
	**High/High (N=257)**	**High/Low (N=73)**	**Low/High (N=47)**	**Low/Low (N=245)**	**None (N=2,478)**	
Male unemployment rate (%)	3.58 (1.24)	3.26 (1.74)	3.82 (1.59)	4.30 (2.28)	3.71 (1.72)	<0.001
**Home Values**						
Median home value ($)	111,995.99 (45,790.69)	67,273.13 (43,866.35)	103,495.56 (38,208.38)	64,082.45 (51,543.65)	83,154.66 (45,910.06)	<0.001
Median rent ($)	550.54 (144.25)	396.55 (94.71)	500.56 (116.24)	386.31 (95.05)	434.01 (115.21)	<0.001
Occupied housing units (%)	87.71 (11.58)	79.53 (12.81)	88.01 (10.33)	81.56 (9.79)	86.21 (8.96)	<0.001
Female-headed households with children but no husband (%)	19.31 (6.02)	18.20 (5.40)	19.05 (4.25)	24.30 (10.02)	20.69 (7.25)	<0.001
**Welfare**						
Households receiving some form of income assistance (%)	41.83 (6.67)	33.15 (9.24)	39.24 (8.09)	25.31 (8.25)	33.27 (9.24)	<0.001
Households receiving welfare assistance (%)	6.34 (2.40)	8.41 (3.70)	6.62 (2.68)	11.39 (5.47)	8.48 (4.12)	<0.001
**Index Measurements**						
Income ICE index	0.1225 (0.1805)	-0.1154 (1589)	-0.0837 (0.1778)	-0.1711 (0.1386)	-0.0592 (0.1604)	<0.001
Race ICE index	0.8649 (0.2090)	0.8723 (0.0909)	0.8705 (0.1990)	0.6347 (0.3563)	0.7985 (0.2647)	<0.001
Race and income ICE index	0.2865 (0.1154)	0.1742 (0.0820)	0.2664 (0.1062)	0.0914 (0.1482)	0.1696 (0.1158)	<0.001

eTable 6: County Demographics by Cluster Patterns for Endoscopy in the United States from 2004-2007 vs. 2008-2010.						
**County Demographic (Mean ± SD)**	**Cluster Pattern (N=Number of Counties)**					***Anova***
	**High/High (N=236)**	**High/Low (N=70)**	**Low/High (N=49)**	**Low/Low (N=236)**	**None (N=2,509)**	
**Population**						
Total in the county (N)	188,211.17 (267,905.41)	36,720.95 (96,117.81)	278,359.20 (749,190.73)	39,980.15 (123,624.92)	82,059.49 (290,570.52)	<0.001
Children (< 18) (%)	25.12 (2.58)	25.18 (3.84)	25.02 (2.43)	27.68 (4.25)	25.30 (3.03)	<0.001
**Race**						
Identifies as Black (%)	7.03 (11.52)	3.63 (7.68)	7.07 (10.69)	9.78 (19.85)	9.47 (14.47)	0.002
Identifies as White (%)	89.05 (13.07)	87.45 (11.61)	89.32 (12.46)	76.06 (19.81)	86.13 (15.60)	<0.001
Foreign-born (%)	4.59 (5.00)	3.99 (4.20)	4.09 (4.42)	6.09 (7.27)	3.02 (4.41)	<0.001
**Education for Adults (25+ years)**						
At least 8 years of education (%)	5.49 (2.29)	10.65 (6.02)	5.86 (2.39)	14.59 (7.57)	8.96 (4.73)	<0.001
< 8 years of education and < high school (%)	10.23 (3.45)	14.66 (4.16)	11.11 (3.26)	15.82 (4.32)	13.69 (4.65)	<0.001
Completed high school (%)	33.40 (7.13)	33.38 (5.76)	35.02 (7.29)	30.97 (5.66)	35.25 (6.44)	<0.001
Bachelor’s degree or higher (%)	23.81 (10.07)	14.95 (6.47)	21.65 (8.75)	14.22 (6.07)	15.91 (7.23)	<0.001
**Income (N or %)**						
Median household income ($)	52,939.71 (12,270.18)	36,412.66 (6,384.67)	51,993.71 (10,465.03)	33,842.37 (7,123.52)	41,669.34 (8,623.15)	<0.001
Below the poverty line (%)	9.05 (3.66)	16.78 (5.47)	8.88 (3.00)	21.51 (8.29)	14.00 (5.94)	<0.001
Unemployment rate (%)	2.73 (1.31)	3.83 (1.82)	2.63 (1.19)	5.05 (2.95)	3.29 (1.73)	<0.001

eTable 6: County Demographics by Cluster Patterns for Endoscopy in the United States from 2004-2007 vs. 2008-2010.						
**County Demographic (Mean ± SD)**	**Cluster Pattern (N=Number of Counties)**					***Anova***
	**High/High (N=236)**	**High/Low (N=70)**	**Low/High (N=49)**	**Low/Low (N=236)**	**None (N=2,509)**	
Male unemployment rate (%)	3.53 (1.27)	3.33 (1.68)	3.81 (1.49)	4.34 (2.30)	3.71 (1.71)	<0.001
**Home Values**						
Median home value ($)	112,113.62 (47,565.53)	67,848.39 (39,095.70)	109,690.91 (39,473.51)	63,287.45 (50,592.60)	83,233.04 (45,841.54)	<0.001
Median rent ($)	552.89 (149.53)	398.53 (91.54)	525.73 (118.66)	376.70 (92.12)	434.79 (114.25)	<0.001
Occupied housing units (%)	88.45 (10.52)	78.77 (13.32)	88.23 (12.19)	81.90 (9.17)	86.12 (9.10)	<0.001
Female-headed households with children but no husband (%)	19.45 (6.32)	18.82 (5.71)	19.30 (4.94)	23.39 (9.29)	20.73 (7.34)	<0.001
**Welfare**						
Households receiving some form of income assistance (%)	41.58 (7.25)	31.87 (9.20)	40.25 (6.32)	25.24 (8.15)	33.38 (9.24)	<0.001
Households receiving welfare assistance (%)	6.29 (2.40)	9.01 (3.77)	6.26 (2.24)	12.16 (5.95)	8.38 (3.97)	<0.001
**Index Measurements**						
Income ICE index	0.1258 (0.1857)	-0.1506 (0.1343)	0.1185 (0.1647)	-0.1888 (0.1415)	-0.0566 (0.1578)	<0.001
Race ICE index	0.8459 (0.2261)	0.8636 (0.1520)	0.8496 (0.2113)	0.6954 (0.3423)	0.7959 (0.2670)	<0.001
Race and income ICE index	0.2846 (0.1229)	0.1504 (0.0701)	0.2792 (0.1063)	0.1024 (0.1361)	0.1697 (0.1173)	<0.001

eTable 7: County Demographics by Cluster Patterns for Fecal Occult Blood Test in the United States from 2004-2007 vs. 2017-2019.						
**County Demographic (Mean ± SD)**	**Cluster Pattern (N=Number of Counties)**					***Anova***
	**High/High (N=85)**	**High/Low (N=85)**	**Low/High (N=93)**	**Low/Low (N=97)**	**None (N=2,740)**	
**Population**						
Total in the county (N)	59,377.65 (99,524.88)	236,296.60 (315,256.81)	106,208.34 (380,422.00)	198,071.18 (582,253.58)	82,637.28 (278,192.58)	<0.001
Children (< 18) (%)	25.75 (3.72)	25.35 (3.28)	24.93 (2.57)	25.41 (3.57)	25.48 (3.17)	0.56
**Race**						
Identifies as Black (%)	4.05 (9.82)	9.92 (11.24)	6.24 (12.01)	13.13 (13.10)	9.22 (14.92)	<0.001
Identifies as White (%)	86.80 (17.64)	84.06 (14.45)	89.43 (13.59)	82.15 (13.47)	85.65 (16.06)	0.0432
Foreign-born (%)	2.94 (3.35)	6.63 (7.37)	2.83 (4.82)	5.43 (5.27)	3.31 (4.72)	<0.001
**Education for Adults (25+ years)**						
At least 8 years of education (%)	8.15 (4.77)	6.39 (3.32)	12.68 (7.68)	8.03 (3.97)	9.16 (5.22)	<0.001
< 8 years of education and < high school (%)	13.63 (3.92)	10.97 (4.15)	15.83 (3.69)	13.11 (4.40)	13.58 (4.70)	<0.001
Completed high school (%)	34.90 (7.30)	31.39 (6.34)	35.00 (6.54)	31.11 (6.67)	34.91 (6.48)	<0.001
Bachelor’s degree or higher (%)	16.04 (7.06)	24.22 (9.47)	13.16 (5.65)	21.21 (10.51)	16.23 (7.53)	<0.001
**Income (N or %)**						
Median household income ($)	39,669.56 (7,618.57)	52,787.47 (12,153.59)	36,535.81 (9,185.34)	48,935.79 (11,696.51)	41,716.71 (9,445.45)	<0.001
Below the poverty line (%)	16.10 (6.56)	10.25 (5.46)	19.03 (8.70)	11.39 (5.14)	14.19 (6.44)	<0.001

eTable 7: County Demographics by Cluster Patterns for Fecal Occult Blood Test in the United States from 2004-2007 vs. 2017-2019.						
**County Demographic (Mean ± SD)**	**Cluster Pattern (N=Number of Counties)**					***Anova***
	**High/High (N=85)**	**High/Low (N=85)**	**Low/High (N=93)**	**Low/Low (N=97)**	**None (N=2,740)**	
Unemployment rate (%)	4.13 (1.97)	2.71 (1.43)	4.95 (2.58)	2.50 (1.02)	3.37 (1.88)	<0.001
Male unemployment rate (%)	4.76 (1.94)	3.29 (1.16)	4.74 (1.89)	3.28 (1.26)	3.71 (1.74)	<0.001
**Home Values**						
Median home value ($)	94,785.39 (38,978.52)	134,160.51 (111,480.23)	78,006.76 (31,019.64)	105,225.00 (40,192.83)	81,807.25 (43,996.46)	<0.001
Median rent ($)	447.94 (103.37)	576.59 (148.86)	411.77 (104.91)	520.09 (136.26)	434.61 (118.51)	<0.001
Occupied housing units (%)	82.97 (12.19)	89.96 (8.62)	86.07 (6.39)	90.27 (7.44)	85.68 (9.54)	<0.001
Female-headed households with children but no husband (%)	20.84 (4.99)	20.24 (7.70)	21.96 (5.73)	21.66 (7.32)	20.73 (7.53)	0.443
**Welfare**						
Households receiving some form of income assistance (%)	31.86 (8.82)	39.48 (8.65)	27.42 (8.15)	25.03 (9.37)	33.47 (9.58)	<0.001
Households receiving welfare assistance (%)	9.73 (4.18)	6.35 (2.97)	13.01 (7.04)	6.51 (2.54)	8.46 (4.11)	<0.001
**Index Measurements**						
Income ICE index	-0.0857 (0.1509)	0.1291 (0.1855)	-0.1493 (0.1870)	0.0621 (0.1894)	-0.0565 (0.1678)	<0.001
Race ICE index	0.8489 (0.2110)	0.7718 (0.2309)	0.8555 (0.2179)	0.7256 (0.2353)	0.7936 (0.2748)	0.007

eTable 7: County Demographics by Cluster Patterns for Fecal Occult Blood Test in the United States from 2004-2007 vs. 2017-2019.						
**County Demographic (Mean ± SD)**	**Cluster Pattern (N=Number of Counties)**					***Anova***
	**High/High (N=85)**	**High/Low (N=85)**	**Low/High (N=93)**	**Low/Low (N=97)**	**None (N=2,740)**	
Race and income ICE index	0.1770 (0.0922)	0.2703 (0.1360)	0.1535 (0.0956)	0.2201 (0.1386)	0.1715 (0.1246)	<0.001

eFigure 1. Prevalence of Breast Cancer Screening in the United States from 1997-2019 for Mammography.

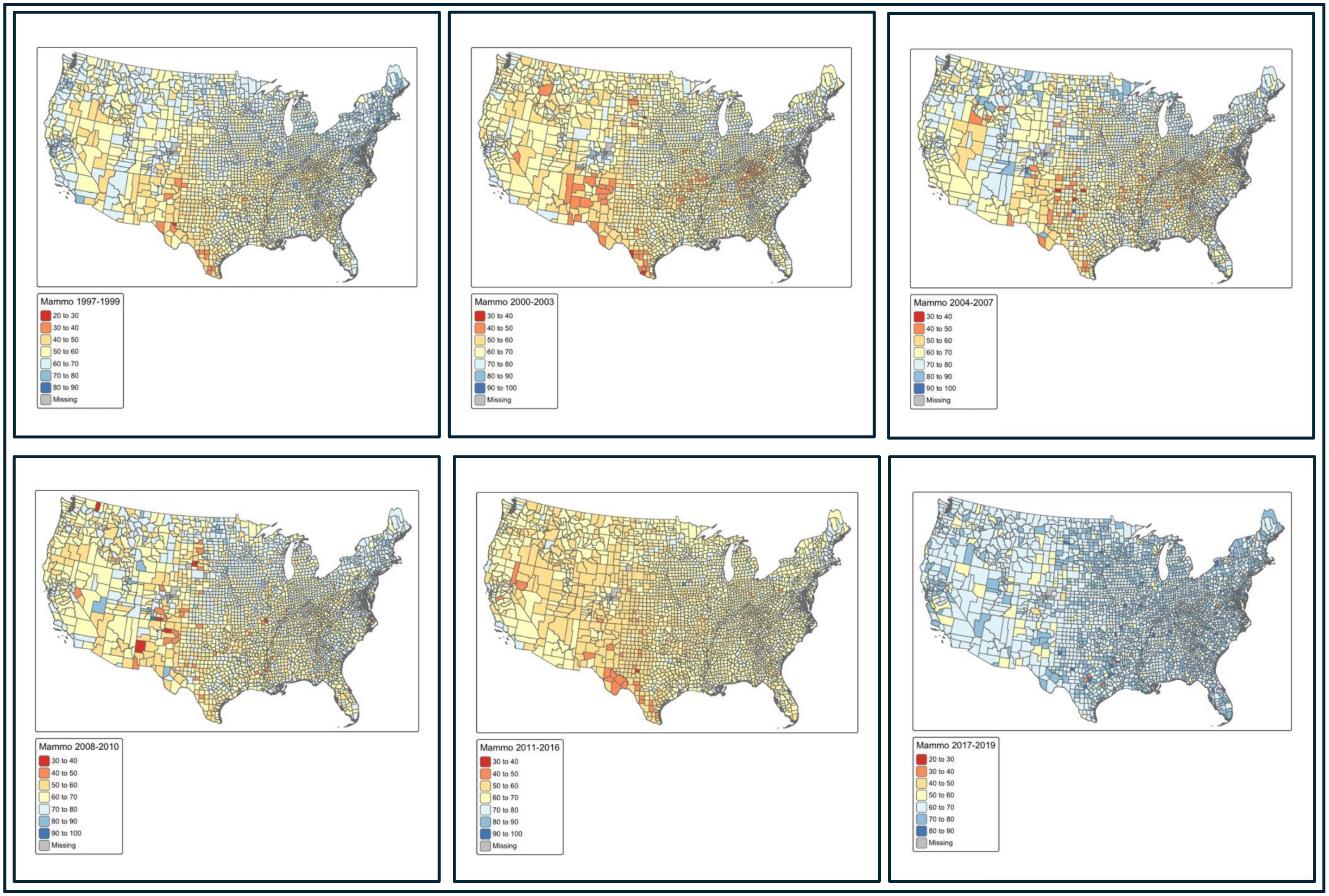

eFigure 2. Prevalence of Cervical Cancer Screening in the United States from 1997-2019 for Pap Smear.

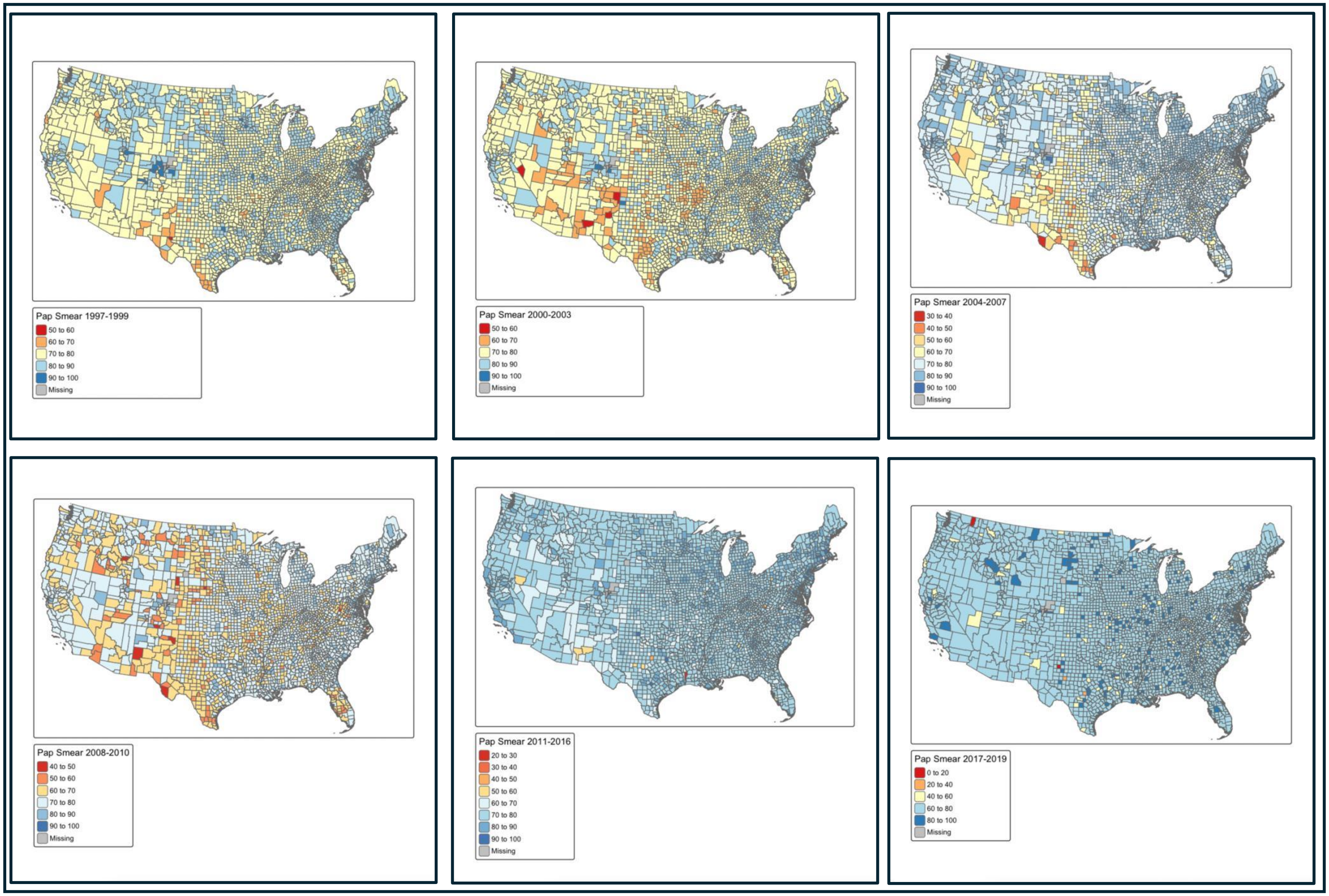

eFigure 3. Prevalence of Colorectal Cancer Screening in the United States from 2004-2019 for Colonoscopy, Colorectal Cancer Test, and Endoscopy.

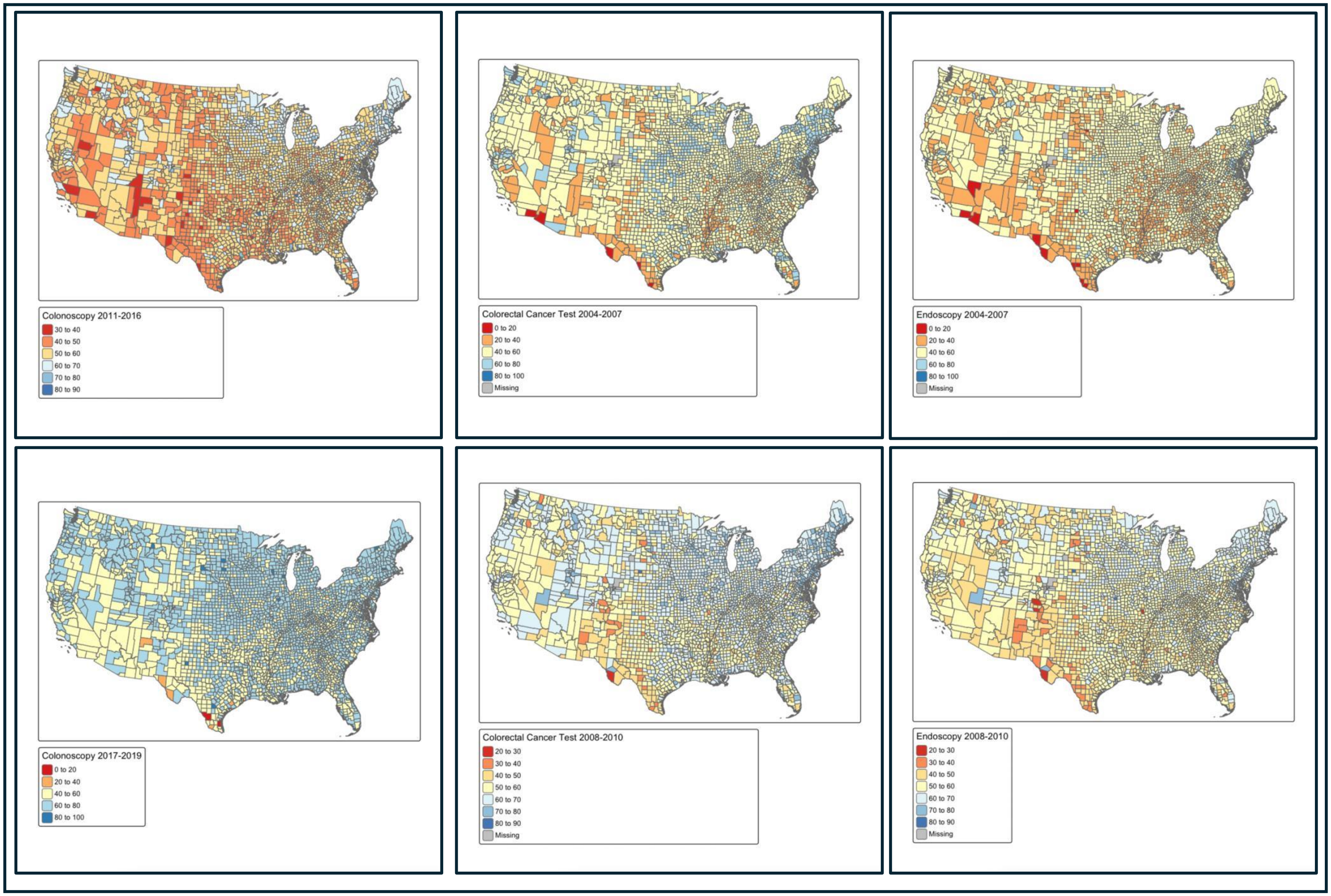

eFigure 4. Prevalence of Colorectal Cancer Screening in the United States from 2004-2019 for Fecal Occult Blood Test.

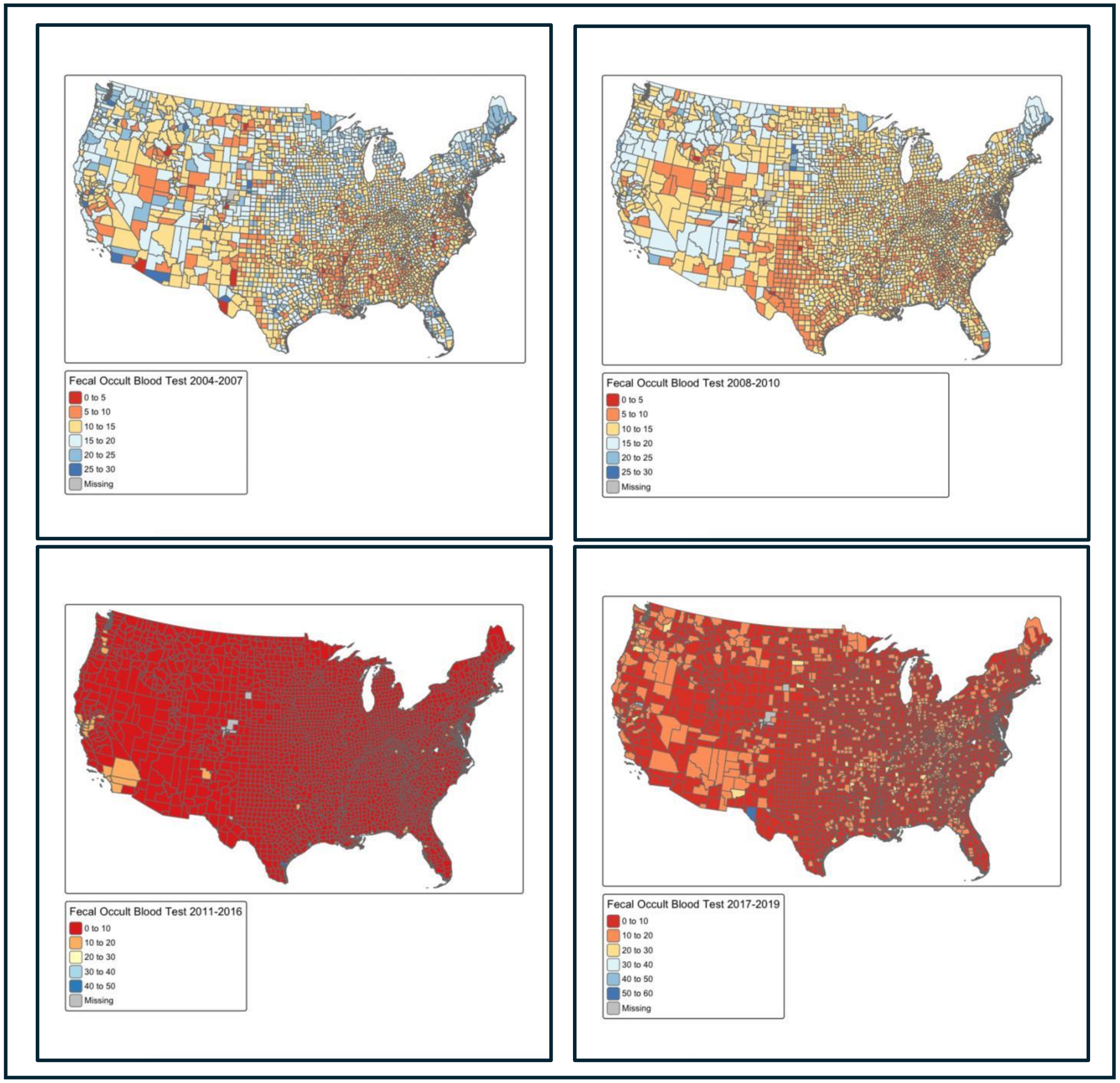

eAppendix. Local Geographical Clusters of County-Level Cancer Screenings in the US

Link to video file

This video shows local geographic clusters of county-level cancer screenings in the US. Spatial clustering techniques identified counties with consistently high (red) or low (blue) screening prevalence and those with shifting trends over time. A, Cluster types based on local mammography for breast cancer screening prevalence and spatial association from 1997 to 2019. B, Cluster types based on Pap test for cervical cancer screening prevalence and spatial association from 1997 to 2019. C, Cluster types based on colonoscopy for colorectal cancer screening prevalence and spatial association from 2011 to 2019. D, Cluster types based on general colorectal cancer test prevalence and spatial association from 2004 to 2010. E, Cluster types based on endoscopy for colorectal cancer screening prevalence and spatial association from 2004 to 2010. F, Cluster types based on fecal occult blood test for colorectal cancer screening prevalence and spatial association from 2004 to 2019. For mammography, Pap test, colonoscopy, general colorectal cancer test, and endoscopy, high-prevalence clusters were largely concentrated in the Northeast, whereas persistent low-prevalence clusters appeared in the Southwest; however, unlike the other screening modalities, no clear or consistent spatial patterns were observed for fecal occult blood test. These spatial patterns—or lack thereof in the case of fecal occult blood test—reflect the complexity of cancer screening uptake and highlight the need for more nuanced public health strategies.
