# Supplementary material for: Geographic and Temporal Patterns of Screening for Breast, Cervical, and Colorectal Cancer in the US, 1997-2019
Source: JAMA Netw Open. 2025 Oct 17;8(10):e2537905. doi: 10.1001/jamanetworkopen.2025.37905 (PMC12534853; doi:10.1001/jamanetworkopen.2025.37905)
Supplement: Supplement 2. — Data Sharing Statement [file jamanetwopen-e2537905-s002.pdf]

Data Sharing Statement

Pradhan. Geographic and Temporal Patterns of Screening for Breast, Cervical, and Colorectal Cancer in the US, 1997-2019. *JAMA Netw Open*. Published October 17, 2025. doi:10.1001/jamanetworkopen.2025.37905

Data
Data available: No

Additional Information
Explanation for why data not available: The data is from the Census and online databases which can be accessed by all.
